# Membrane Characterization by Microscopic and Scattering Methods: Multiscale Structure

**DOI:** 10.3390/membranes1020091

**Published:** 2011-04-13

**Authors:** Rahma Tamime, Yvan Wyart, Laure Siozade, Isabelle Baudin, Carole Deumie, Karl Glucina, Philippe Moulin

**Affiliations:** 1 Laboratoire de Mécanique, Modélisation et Procédés Propres (M2P2–CNRS UMR 6181), Université Paul Cézanne Aix Marseille, Europôle de l'Arbois, 13545 Aix en Provence Cedex 04, France; E-Mails: rahma.tamime@etu.univ-cezanne.fr (R.T.); philippe.moulin@univ-cezanne.fr (P.M.); 2 Institut FRESNEL (UMR 6133), Université Paul Cézanne Aix Marseille, Domaine Universitaire de St Jérôme, 13397 Marseille Cedex 20, France; E-Mails: laure.siozade@fresnel.fr (L.S.); carole.deumie@fresnel.fr (C.D.); 3 Suez Environnement, CIRSEE, Pôle Qualité Eau, 38, rue du Président-Wilson, 78230 Le Pecq, France; E-Mails: isabelle.baudin@suez-env.com (I.B.); karl.glucina@suez-env.com (K.G.)

**Keywords:** membrane, atomic force microscopy, white light interferometry, ellipsometry, roughness

## Abstract

Several microscopic and scattering techniques at different observation scales (from atomic to macroscopic) were used to characterize both surface and bulk properties of four new flat-sheet polyethersulfone (PES) membranes (10, 30, 100 and 300 kDa) and new 100 kDa hollow fibers (PVDF). Scanning Electron Microscopy (SEM) with “in lens” detection was used to obtain information on the pore sizes of the skin layers at the atomic scale. White Light Interferometry (WLI) and Atomic Force Microscopy (AFM) using different scales (for WLI: windows: 900 × 900 μm^2^ and 360 × 360 μm^2^; number of points: 1024; for AFM: windows: 50 × 50 μm^2^ and 5 × 5 μm^2^; number of points: 512) showed that the membrane roughness increases markedly with the observation scale and that there is a continuity between the different scan sizes for the determination of the RMS roughness. High angular resolution ellipsometric measurements were used to obtain the signature of each cut-off and the origin of the scattering was identified as coming from the membrane bulk.

## 1. Introduction

Water treatment by ultrafiltration (UF) can provide permeate quality far beyond the current regulatory requirements for drinking water consumption. Nevertheless, the development of this membrane process is limited by fouling phenomena. The parameters influencing fouling can be classified as follows: membrane chemical parameters (materials, surface charge, hydrophobicity) and structural parameters (porosity, roughness, pore size, pore shape and pore size distribution) as well as water parameters (organic, mineral or biological pollutants, suspended and/or dissolved matter, *etc.*).

To study the structural parameters of the membrane, three techniques are generally used: displacement techniques [1,2], tracers retention techniques [3] and microscopic techniques [1,4]. Among the latter, Scanning Electron Microscopy (SEM) and Atomic Force Microscopy (AFM) are the most used. SEM has been used for various applications, for example, the qualification of the pore nature for a same cut-off [5] and the measurement of the fouling layer [6]. AFM can be used under three modes: contact, non-contact and tapping [7]. This technique provides a high-resolution representation of the surface (1 nm) and gives information such as roughness, pore size, pore density and/or pore size distribution [8].

In this work, four polyethersulfone flat sheet membranes (MWCO = 10–300 kDa) were characterized as well as a 100 kDa PVDF hollow fiber membrane. For this purpose, AFM, White Light Interferometry (WLI) and Ellipsometry were used.

## 2. Experimental Section

In this study, WLI measurements were made using Talysurf CCI 3000 Å. This non-contact surface profiler makes it possible to obtain image sizes from 360 μm × 360 μm to 900 μm × 900 μm with a resolution of 1024 × 1024 points. In the case of the 360 μm × 360 μm scan size, the accessible lateral resolution was 0.5 μm and the accessible vertical resolution was 0.1 Å.

The instrument used for the AFM measurements was a Q-Scope 250 (QUESANT), whose scan tip allows the study of zones ranging from 1 μm × 1 μm to 80 μm × 80 μm with a resolution of 512 × 512 points. The images obtained were numerically processed to extract the roughness parameters.

The optical technique used was the Ellipsometry of Angle Resolved Scattering. It gives access to the polarimetric phase shift and allows the analysis of the fine structure of the light scattering intensity of the wave polarization scattered by the sample (called speckle). Ellipsometry allows the identification of the optical signatures of the various sources of scattering and in particular the difference between the surface and the bulk effects.

## 3. Results and Discussion

In order to study the topography of each membrane and to determine their roughness, two different scan sizes were used for each technique: 900 μm × 900 μm and 360 μm × 360 μm for WLI (1024 points) and 50 μm × 50 μm and 5 μm × 5 μm for AFM (512 points). Measurements were taken at three different sample locations to obtain an average roughness spectrum and a mean value of the roughness. For example, Table 1 presents the different views (top and 90° view) obtained for the 100 kDa membrane.

From these different views, the roughness spectrum can be obtained by Equation (1):

(1) $$\gamma (\vec{\sigma })=\frac{4\pi^{2}}{S}{\left|\tilde{h}(\vec{\sigma })\right|}^{2}$$

where σ = (σ,σ_y_): spatial pulsation, *h̃* : spatial Fourier transform of the h (x, y) profile, S = L^2^ (measured area).

**Table 1:** Images obtained for the 100 kDa flat sheet membrane by White Light Interferometry (WLI) and Atomic Force Microscopy (AFM).

**Technique**	**Scan size**	**Top view**	**90° view**
WLI	900 μm × 900 μm	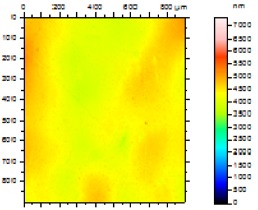	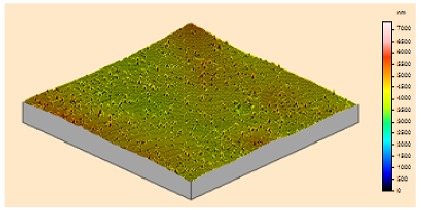
	360 μm × 360 μm	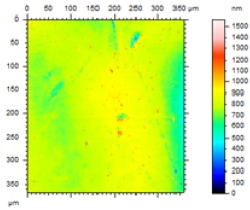	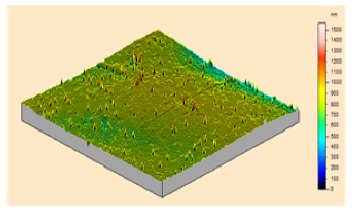
AFM	50 μm × 50 μm	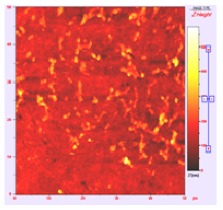	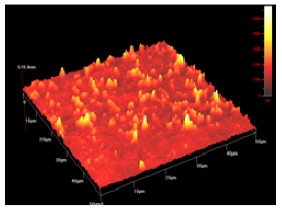
	5 μm × 5 μm	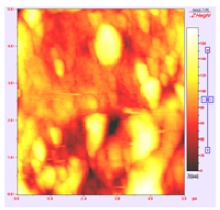	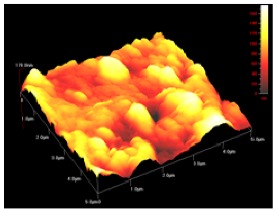

**Figure 1:**
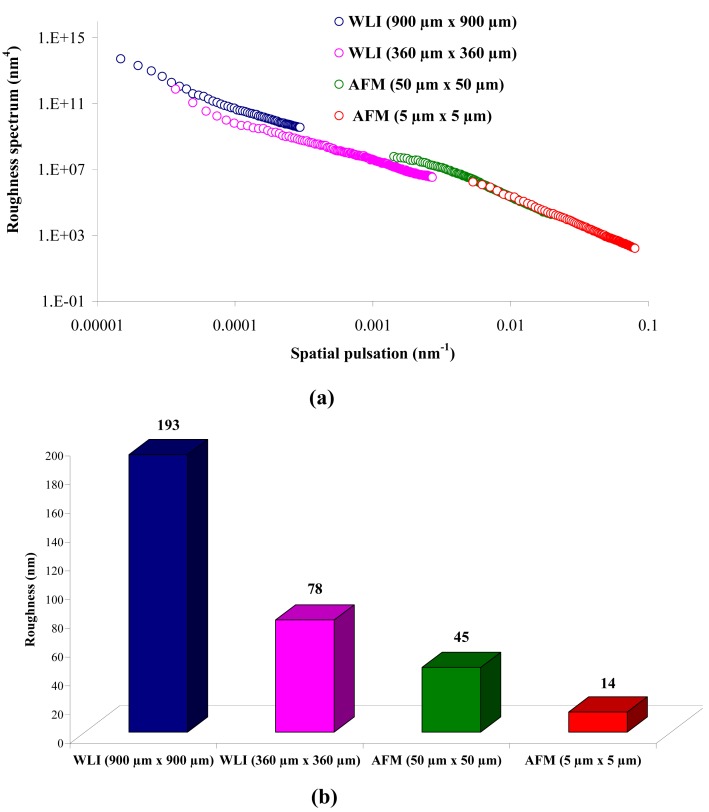
Roughness spectrum **(a)** and evolution of the roughness **(b)** for the 100 kDa flat sheet membrane by White Light Interferometry (WLI) and Atomic Force Microscopy (AFM).

From Figure 1 (a), it can be observed that there is a continuity in the determination of the roughness when the measurement technique and the scan size change (from WLI in 900 μm × 900 μm to AFM in 5 μm × 5 μm), and when the scale changes for measurements using the same technique (from a 50 μm × 50 μm to a 5 μm × 5 μm scan size for AFM). From Figure 1 (a), it is possible to determine the RMS roughness R_q_ of the membrane for each technique, whatever the scan size, according to Equation (2):

(2) $${R_{q}}^{2}=\int_{\vec{\sigma }}\gamma (\vec{\sigma })d\vec{\sigma }=2\pi \int_{\sigma }\sigma \bar{\gamma }(\sigma )d\sigma$$

**Figure 2:**
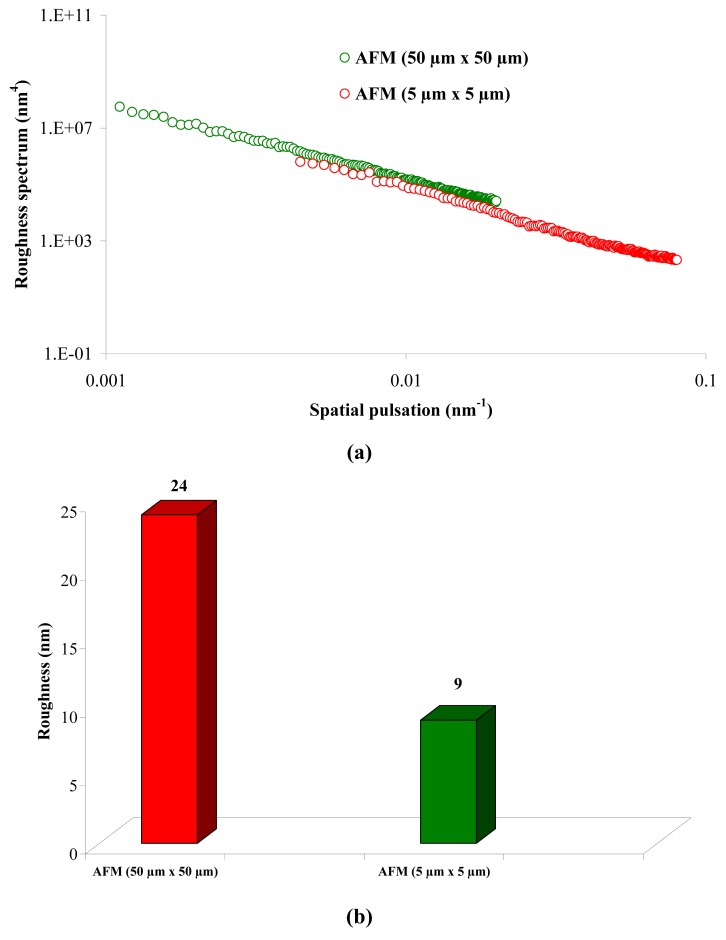
Roughness spectrum **(a)** and evolution of the roughness **(b)** for the hollow fiber (100 kDa, PVDF) by AFM for 50 μm × 50 μm and 5 μm × 5 μm windows.

Figure 1 (b) shows that the value of the roughness depends on the observation scale: the higher the scan size, the higher the roughness value. In that case, RMS roughness values must always be given with the associated scan size. Whatever the geometry, the material and the MWCO in the range studied, similar results must be obtained: for example, the results for the UF hollow fiber (100 kDa, PVDF) are presented in Figure 2.

**Figure 3:**
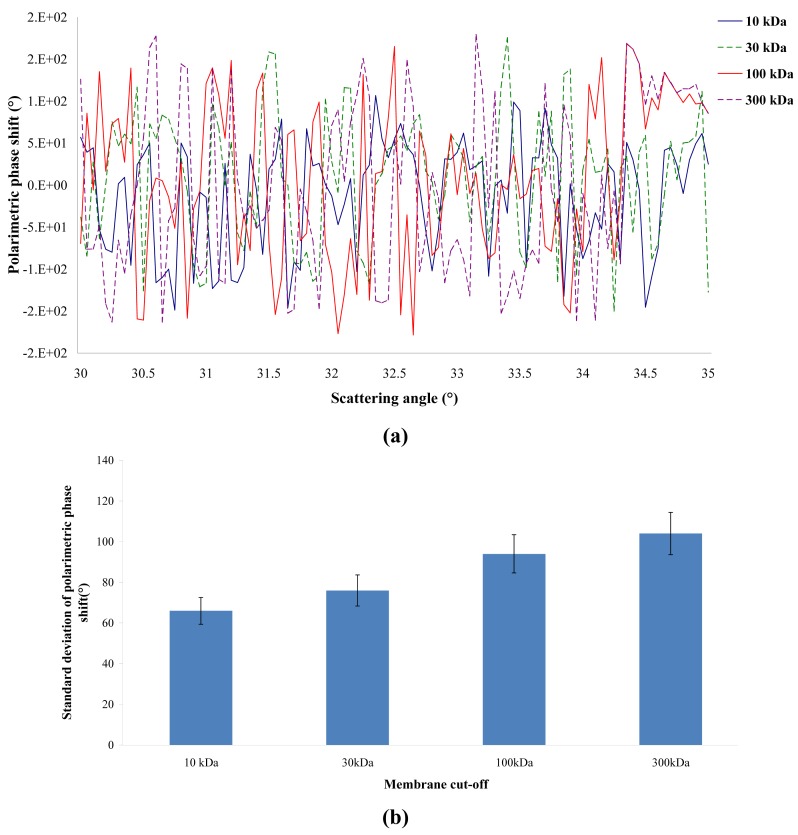
Polarimetric phase shift **(a)** and standard deviation of the polarimetric phase shift **(b)** as a function of the scattering angle.

The polarimetric behavior of the four membranes was studied by ellipsometry of angle resolved scattering at high resolution. Figure 3 (a) shows the variation of the polarimetric phase shift as a function of the scattering angle. It can be observed that the polarimetric phase shift varies very quickly between −π and π. This is the typical behavior of a bulk scattering material [9]. It seems that the ellipsometric technique can provide information allowing us to conclude whether bulk effects have more importance than surface effects. It is also possible to plot the standard deviation of the polarimetric phase shift as a function of the scattering angle (Figure 3 (b)). It can be observed that the higher the pore size, the higher the standard deviation of the polarimetric phase shift. This result means that ellipsometry is able to differentiate several membranes according to their MWCO.

## 4. Conclusions

WLI and AFM are very useful techniques for the characterization of the surface roughness of organic membranes. The combined use of these two techniques at different observation scales offers a multi-scale analysis of membrane surface roughness. AFM and WLI have different scan sizes: AFM exhibits smaller scan sizes than WLI. This difference makes these two techniques complementary to each other. AFM and WLI show that the determination of the membrane roughness depends on the observation scale. The roughness of a membrane increases remarkably with the observation scale. The present results show that the speckle of the scattered wave is sufficient to allow differentiation between membranes. The polarization analysis clearly shows that the light scattering comes essentially from the membrane bulk. The angular variations of the polarimetric phase shift increase with the porosity and allow discrimination among different membrane porosities.
